# Sociodemographic and Psychosocial Factors Influencing COVID-19 Testing Uptake: Insights from Urban and Rural Communities in South Africa

**DOI:** 10.4269/ajtmh.23-0810

**Published:** 2025-02-04

**Authors:** Nokhanyo Xaba, Onaiza Qureshi, Aneeta Pasha, Amyn Malik, Anne Hoppe, Zaw Myo Tun, National Fynn, Goodman Sibeko, Saira Khowaja, Aamir Javed Khan

**Affiliations:** ^1^ Interactive Research & Development (IRD), South Africa, Durban, South Africa;; ^2^ Interactive Research & Development (IRD), Pakistan, Karachi, Pakistan;; ^3^ Interactive Research & Development (IRD) Global, Singapore;; ^4^ FIND, Geneva, Switzerland;; ^5^ Elizabeth Glaser Pediatric AIDS Foundation, Geneva, Switzerland;; ^6^ Department of Psychiatry, University of Cape Town, Cape Town, South Africa

## Abstract

Access, demand, and acceptance of coronavirus disease 2019 (COVID-19) testing have varied globally. This study explored the sociodemographic and psychosocial risk factors that contribute to the uptake of COVID-19 testing in community settings in South Africa. This paper presents a cross-sectional secondary analysis using data from a cluster randomized controlled trial and a nested perception survey of COVID-19 antigen testing in communities located in urban (eThekwini, KwaZulu-Natal) and rural settings (Worcester, Eastern Cape) in South Africa. Individuals who were reluctant to get tested participated in the perception survey. Data were analyzed using descriptive statistics and multivariable logistic regression to assess linear associations and estimate adjusted odds ratios (ORs). The analysis was conducted on 3,074 individuals, of whom 2,509 (81.6%) provided consent for COVID-19 testing. Among those, 2,505 (81.5%) tested negative, and 4 (0.1%) tested positive for COVID-19. The mean age of participants was 38 (SD = 14.61), and 57% were male. Females (OR: 1.27; 95% CI = 1–1.6), individuals older than 56 years (OR: 1.95; 95% CI = 1.24–3.07), and those who were vaccinated (OR: 1.99; 95% CI = 1.53–2.60) were more likely to consent. Individuals who had previously tested positive for severe acute respiratory syndrome coronavirus 2 were less likely to consent to testing (OR: 0.64; 95% CI = 0.11–0.46). No link was found between depression, anxiety, substance use, and willingness to undergo COVID-19 testing. A perceptions survey involving 704 participants, which explored factors influencing testing willingness, found that older adults, and urban populations were less likely to undergo COVID-19 testing. Targeted health campaigns may improve testing rates. Larger-scale implementation research is required to explore best practices for improving testing rates and confidence in population-level detection within South Africa.

## INTRODUCTION

Coronavirus disease 2019 (COVID-19) has impacted almost all regions of the world since its outbreak in December 2019, including South Africa.^1^^,^^2^ In December 2021, South Africa accounted for 36.7% of recorded COVID-19 cases and 42.3% of recorded COVID-19 deaths on the continent.^2^ Although all South African citizens were affected by the sudden and unprecedented spread of the pandemic, structural inequality, poverty, unemployment, and a lack of access to quality health care and other services disproportionately impacted the poor and vulnerable.^3^^–^^5^

During the height of the pandemic, curbing the transmission of COVID-19 through testing, effective isolation of cases, and contact tracing was a public health priority. In early 2020, many African governments quickly increased COVID-19 surveillance and control measures, drawing on lessons learned from previous outbreaks of other infectious diseases. Laboratory testing was thus an essential component of the COVID-19 outbreak response. However, according to Madhi and Nel,^2^ the burden of COVID-19 was likely underreported in most African countries because of insufficient diagnostic capacity. South Africa reported the highest testing rate (248 per 1,000 population), which is, nevertheless, one-fourteenth of the testing performed in high-income countries.^2^^,^^6^ In South Africa, an ambitious community testing program was implemented which aimed to identify infected individuals, quarantine them, and trace their contacts.^4^ However, as highlighted by Mendelson and Madhi (2020), the effectiveness of this program was hampered by systemic issues within the testing strategy, including inadequate testing capacity and low testing rates relative to the number of COVID-19 cases. To improve outcomes, it was essential to enhance the availability of testing in underserved communities, engage the public through clear health messaging, and integrate COVID-19 testing with existing health services. By addressing these challenges, South Africa can better use its community testing program to effectively manage the pandemic.^7^

During the COVID-19 pandemic, the implications of changes in daily life, loss of income, and the death of close family members increased anxiety and depression among South Africans. Small rural-based studies in South Africa found a prevalence of depression of 27%, and other research found a prevalence of depression of 25.2% in urban settings.^8^^,^^9^ These rates are higher than pre-pandemic estimates. For instance, before the pandemic, substance misuse trends in South Africa indicated lower prevalence rates for mental health issues and substance abuse. According to Pasche and Myers (2012), substance misuse trends in South Africa before the pandemic showed different patterns. Similarly, Peltzer, Davids, and Njuho (2011) reported that drinking problems in South Africa were significant but did not reach the same levels as during the pandemic.^8^^,^^9^ Additionally, alcohol remains the primary substance of abuse in South Africa with an estimated 7.5–31.5% of South Africans having or being at risk of developing an alcohol problem during the COVID-19 pandemic. This observation is consistent with the increased stress and mental health burdens observed during this period. However, this range is broader than pre-pandemic estimates, indicating a potential increase in alcohol-related issues due to the pandemic’s impact.^10^^,^^11^ Given the heightened mental health burdens and substance abuse issues observed during the pandemic, it is crucial to integrate mental health and substance abuse screening and counseling into COVID-19 testing and contact tracing efforts. This approach can help address the holistic needs of individuals, particularly in marginalized communities where access to mental health services is limited. By analyzing existing data on depression, anxiety, and substance abuse screening, along with COVID-19 testing behaviors and perceptions from a randomized controlled trial (RCT) conducted in South Africa, we aimed to explore psychosocial factors that influenced people’s likelihood of testing for COVID-19. This inquiry can support actionable evidence for policymakers and health authorities, informing the development of targeted interventions to mitigate the mental health and substance abuse impacts of potential future pandemics.

The perceptions study explored the factors that influenced people’s likelihood of testing for COVID-19. Our aim was to better understand the sociocultural influences on poor testing and treatment behavior and to strengthen the evidence for community-based testing strategies in diverse populations.

## MATERIALS AND METHODS

### Study design and secondary data collection.

This was a retrospective cross-sectional analysis using secondary data from the following two sources:

1. A cluster RCT of COVID-19 mass screening was conducted at nine community camps in Durban and Worcester. The following variables were extracted from the trial dataset:
  - Participant characteristics, including sociodemographic information, and COVID-19 exposure risk/contact history (such as the number of household members, daily interactions, usage of public transport, and participation in social or religious gatherings);
  - COVID-19 vaccine history (vaccination against COVID-19 and names of vaccines);
  - COVID-19 test: consent or refusal for testing and reasons for refusal;
  - Symptoms of depression (Patient Health Questionnaire, 9-Item);^12^
  - Symptoms of anxiety (Generalized Anxiety Disorder Scale, 7-Item);^13^
  - Reported alcohol use (Alcohol Use Disorders Identification Test, 10-Item);
  - Reported drug use (Drug Use Identification Test, 11-item).^14^
2. A perceptions study nested within the RCT explored the sociocultural factors influencing people’s likelihood to test for COVID-19. As a result of the high COVID-19 screening and testing refusal rates within the RCT, a parallel perceptions study was implemented to investigate the factors associated with the low uptake of COVID-19 services. The following factors were extracted from the perceptions study:
  - Knowledge about COVID-19;
  - Perceptions of COVID-19 testing;
  - Perceptions of COVID-19 vaccination;
  - Help-seeking behaviors related to COVID-19 and the associated risk of mental health disorders.

The findings of the intervention evaluation from the RCT are not presented in this paper.

### Sample and setting.

Participants included individuals recruited for the RCT and the perceptions study. They were from farming and peri-farming communities in the Cape Winelands, as well as from multiple households in densely populated areas and public spaces, such as public transport ranks and shopping malls, in townships within the eThekwini District, Kwa-Zulu Natal. Individuals who expressed interest in participating in the study were approached for informed consent and were administered the COVID-19 and mental health screening tools through mobile health platforms such as *Hydra* (Interactive Health Solutions, Karachi, Pakistan; https://www.ihsinformatics.com/products/) and Open Data Kit (https://getodk.org/). If an individual reported symptoms of COVID-19 or recent exposure to the virus, a free antigen rapid diagnostic test (Ag-RDT) was provided, along with baseline and endline mental health and substance abuse screenings. Individuals who screened positive for COVID-19 on the Ag-RDT were enrolled in either the intervention or control arm of the RCT, referred for a polymerase chain reaction test, and given information on isolation and household contact referrals. Individuals who refused COVID-19 testing were invited to participate in a survey regarding their views on COVID-19 screening and testing (“perception survey”). Only those participants who refused to screen for COVID-19 were enrolled in and administered the perception survey.

## STATISTICAL ANALYSES

The raw data collected for this study were cleaned before being imported into SPSS 26 (IBM Corp., Armonk, NY) for data analysis. Cut-off scores for standardized scales were used to tabulate categories for depression through Patient Health Questionnaire 9-item scale^12^ (5–9, 10–14, 15–19, and 20–27 to categorize the severity of depression as “mild,” “moderate,” “moderately severe,” and “severe”), anxiety through Generalized Anxiety Disorder 7-item scale^13^ (5–9, 10–14, 15–19, and 20–27 for “mild,” “moderate,” “moderately severe,” and “severe” anxiety), alcohol use risk through Alcohol Use Disorders Identification Test (0–7, 8–15, 16–19, and 20–40 for “Low,” “Medium,” “High,” and “Very High” risk), and drug use through Drug Use Disorders Identification Test (0–24 and more than 25 for “Low Risk” and “At Risk”).^14^

Descriptive statistics were used to summarize the characteristics of the sample, including mean, SD, frequency, and proportion. Multivariable logistic regression was used to examine the association between willingness to test for COVID-19 and demographic variables, COVID-19 characteristics, and mental health symptoms. Adjusted odds ratio calculations were conducted to assess the magnitude of the association between these variables.

In this study, missing data are defined as instances in which key sociodemographic information or responses to the screening and perceptions form were absent. We chose to retain all participants in the analysis, regardless of missing data on certain variables, to maximize the utilization of existing data.

## RESULTS

**Figure 1.**
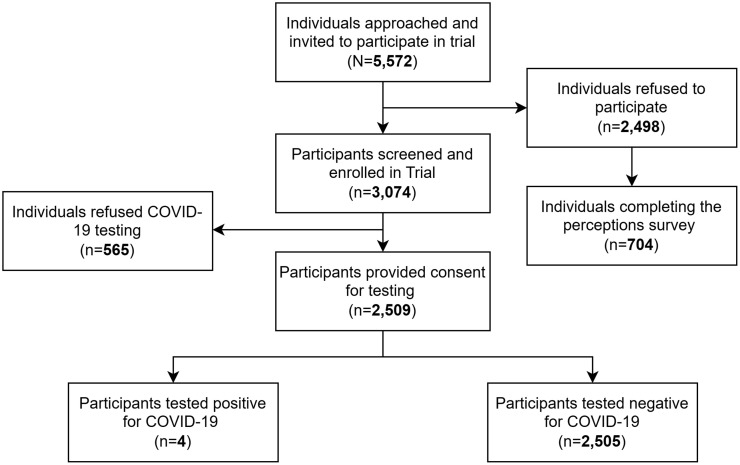
Study diagram for the randomized controlled trial.

Data were analyzed for a total of 3,074 individuals who were screened as part of the RCT and 704 individuals who completed the COVID-19 perceptions survey. Of these, 2,509 (81.6%) provided consent for COVID-19 testing and 2,505 (81.5%) tested negative (the screening and testing consort shown in Figure 1). Only four (0.1%) individuals were found to be positive for COVID-19.

The predominant age group among participants was between 26 and 35 (32% of the population), with a mean age of 38 (Mean = 38.04; SD = 14.61). Male participants (57%; *n* = 1,746) outnumbered females (39%; *n* = 1,197) and individuals of other genders (0.1%, *n* = 3). A significant proportion of participants lived in multi-family member households, with 78% living in households of two to six family members, 15.5% living with more than seven family members, and only 13 (0.4%) individuals living alone. Regarding public interactions, defined as the individual’s number of physical interactions with people on a daily basis, 43% of participants interacted with fewer than 14 individuals daily, whereas 53% interacted with more than 15 people daily. Moreover, 2,404 (78%) participants used public transport, and 62% reported attending large social or religious gatherings. Similar characteristics were observed in participants from the perceptions survey in terms of age (38% between the ages of 26 and 35; M = 36.44; SD = 12.60) and sex (61% male and 38% female) (Table 1).

**Table 1:** Sociodemographic characteristics of participants from the RCT and perception survey

Variables		RCT Participants (*N* = 3,074)		Perception Survey (*N* = 704)	
		*n*	%	*n*	%
City	Durban	2,276	74%	273	39%
	Worcester	798	26%	431	61%
Sex	Males	1,746	57%	435	61%
	Females	1,197	39%	266	38%
	Other	3	0.1%	3	0.4%
	Not reported	128	4%	–	–
Age	18–25 years	617	20%	131	19%
	26–35 years	998	32%	268	38%
	36–45 years	580	19%	154	22%
	46–55 years	370	12%	79	11%
	>56 years	451	15%	72	10%
	Not reported	58	2%	–	–
Household size	Lives alone	13	0.4%	–	–
	Up to 3 family members	1,125	36.6%	–	–
	Between 4 and 6 family members	1,272	41.4%	–	–
	Between 7 and 9 family members	336	10.9%	–	–
	>10 family members	139	4.5%	–	–
	Not reported	189	6.1%	–	–
Daily interactions with people	≤5 people	401	13%	–	–
	Between 6 and 14 people	913	30%	–	–
	≥15 people	1,616	53%	–	–
	Not reported	144	5%	–	–
Public transport usage	Uses public transport	2,404	78%	–	–
	Does not use public transport	579	19%	–	–
	Not reported	91	3%	–	–
Attendance at large religious gatherings	Yes	1,898	62%	–	–
	No	1,075	35%	–	–
	Not reported	101	3%	–	–

RCT = randomized controlled trial.

### Willingness to test and characteristics of COVID-19 service use.

Participant characteristics related to COVID-19, including previous testing, exposure, symptoms, vaccination status, and sociodemographic variables for those who consented, are presented in Table 2.

**Table 2:** COVID-19 characteristics of patients by willingness to test for the disease in the randomized controlled trial

Characteristics	Durban		Worcester	
	Refused to Test (*n*, %)	Agreed to Test (*n*, %)	Refused to Test (*n*, %)	Agreed to Test (*n*, %)
Age in years (*N* = 2,977)				
18–25	106, 21%*	351, 32%*	0	151, 19%
26–35	213, 43%*	553, 33%*	0	215, 27%
36–45	103, 21%*	299, 18%*	1, 100%	169, 21%
46–55	45, 9%*	181, 11%*	0	139, 18%
56 and older	35, 7%*	298, 18%*	0	118, 15%
Gender (*N* = 3,015)				
Male	333, 64%	1,011, 60%	0	53, 7%
Female	175, 36%	644, 38%	2, 100%	379, 48%
Other	0	0	0	3, 0.4%
Previous COVID-19 testing (*N* = 2,917)				
Did not test positive previously	422, 86%	1,453, 88%	1, 50%	550, 71%
Tested positive previously	68, 14%	202, 12%	1, 50%	220, 29%
Self-reporting of exposure to persons with COVID-19 (*N* = 2,840)				
Unexposed	445, 98%	1,565, 97%	2, 100%	735, 95%
Exposed	9, 2%	46, 3%	0	38, 5%
COVID-19 symptoms in the last 48 hours (*N* = 3,015)				
No symptoms	476, 91%	1,526, 90%	2, 100%	734, 92%
Symptoms present	46, 9%	171, 10%	0	60, 8%
Vaccination status (*N* = 2,937)				
Not vaccinated	371, 77%*	1,027, 61%*	2, 100%	312, 40%
Vaccinated	108, 23%*	644, 39%*	0	473, 60%
Depression and anxiety symptoms (*N* = 2,989)				
Reported no symptoms	132, 25%	447, 26%	1, 50%	383, 49%
Reported symptoms	389, 75%	1,245, 74%	1, 50%	391, 51%
Substance use risk (*N* = 2,985)				
Reported no risk	350, 67%^†^	1,230, 73%^†^	2, 100%	476, 62%
Reported at risk	170, 33%^†^	460, 27%^†^	0	297, 38%

COVID-19 = coronavirus disease 2019.

* *P*-value <0.001.

^†^ *P*-value <0.05.

### Willingness to test and mental health symptoms.

**Figure 2.**
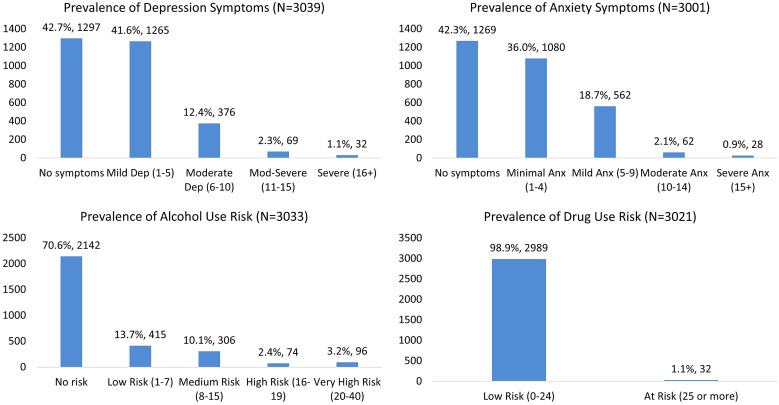
Pooled prevalence rates for depression and anxiety symptoms and alcohol and drug use risk.

A review of mental health screening responses at baseline identified high prevalence rates for depression and anxiety among the participants. More than 57% of participants exhibited depressive symptoms, 58% exhibited anxiety symptoms, and 29% were at risk for alcohol use (pooled prevalence rates are shown in Figure 2). The risk of drug use was low, with 1% classified as high risk and 99% classified as low risk.

When exploring the link between participants’ willingness to test and their mental health status, the study found a slightly higher severity of depression (χ^2^(df [degree of freedom]) = 12.689; *P* <0.05) and anxiety symptoms (χ^2^(df) = 24.317; *P* <0.001) among people who refused to test for COVID-19 compared with those who had no symptoms (Table 3). Although there was a similar increased proportion of refusal to test among people who scored low (20%) and medium risk (19%) for alcohol abuse, this association was not found to be significant for either substance use outcome (alcohol or drug use).

**Table 3:** Proportion of individuals with symptoms of depression and anxiety and risk for alcohol and drug use in the screened population in the randomized controlled trial

Mental Health Outcomes	Symptom	Refused to Test (*n*, %)	Agreed to Test (*n*, %)	Pearson χ^2^	df	*P*-Value
Depression screening (patient health questionnaire, nine-item)	No symptoms	190, 15%	1,102, 85%	12.689	4	<0.05
	Mild depression (1–5)	239, 19%	991, 81%			
	Moderate depression (6–10)	75, 21%	289, 79%			
	Moderate to severe (11–15)	12, 18%	53, 82%			
	Severe (16+)	5, 16%	26, 84%			
Anxiety screening (general anxiety disorder scale, seven-item)	No symptoms	175, 14%	1,079, 86%	24.317	4	<0.001
	Minimal anxiety (1–4)	215, 20%	837, 80%			
	Mild anxiety (5–9)	113, 21%	438, 79%			
	Moderate anxiety (10–14)	7, 11%	55, 89%			
	Severe anxiety (15+)	2, 7%	25, 93%			
Alcohol use screening (alcohol use disorders identification test)	No risk	355, 17%	1,753, 83%	7.223	5	>0.05
	Low risk (1–7)	80, 20%	325, 80%			
	Medium risk (8–15)	58, 19%	241, 81%			
	High risk (16–19)	11, 15%	61, 85%			
	Very high risk (20–40)	18, 19%	75, 81%			
Drug use screening (drug use identification test)	Low risk (0–24)	512, 17%	2,424, 83%	0.387	2	>0.05
	At risk (25 or more)	4, 13%	26, 87%			

df = degree of freedom.

### Risk factors associated with consent for COVID-19 testing.

An exploration of other factors influencing participants’ consent to COVID-19 testing (Table 4) showed that among the participants, individuals aged 56 and older were twice as likely to consent to testing (OR: 1.95; 95% CI = 1.24–3.07; *P* <0.001). Women were 1.27 times more likely to consent (OR: 1.27; 95% CI = 1–1.6; *P* <0.05). Those who were vaccinated were 1.99 times more likely to consent (OR: 1.99; 95% CI = 1.53–2.60; *P* <0.001), whereas individuals who had previously tested positive for COVID-19 were 0.64 times less likely to consent to testing (OR: 0.64; 95% CI = 0.11–0.46; *P* <0.001). Depression and anxiety symptoms, as well as substance use risk, were not found to be significantly associated with adjusted odds for willingness to test.

**Table 4:** Odds ratio (logistic regression) results of predictors for willingness to test for COVID-19 in the randomized controlled trial

Predictor	Characteristic	Standard Error	Odds Ratio	95% CI Lower	95% CI Upper	*P*-Value
Age	18–25	–	1	–	–	–
	26–35	0.115	0.767	0.572	1.028	0.075
	36–45	0.148	0.847	0.601	1.193	0.341
	46–55	0.214	0.999	0.656	1.521	0.994
	>56	0.452	1.954	1.241	3.075	0.004
Sex	Males	–	1	–	–	–
	Females	0.15	1.269	1.007	1.6	0.044
City	Durban	–	1	–	–	–
	Worcester	186.376	185.602	25.931	1,328.419	0.000
Exposure to COVID-19	No exposure	–	1	–	–	–
	Has had exposure	0.41	1.07	0.505	2.266	0.86
Vaccination status	Not vaccinated	–	1	–	–	–
	Vaccinated	0.269	1.989	1.526	2.593	0
Previous COVID-19 testing	Did not test positive previously	–	1	–	–	–
	Tested positive previously	0.107	0.644	0.464	0.892	0.008
Depression and anxiety symptoms	No symptoms reported	–	1	–	–	–
	Symptoms reported	0.155	1.223	0.954	1.567	0.113
Substance use risk	No risk reported	–	1	–	–	–
	Risk reported	0.119	0.959	0.753	1.222	0.737

COVID-19 = coronavirus disease 2019.

### Perceptions regarding willingness to test for COVID-19.

**Figure 3.**
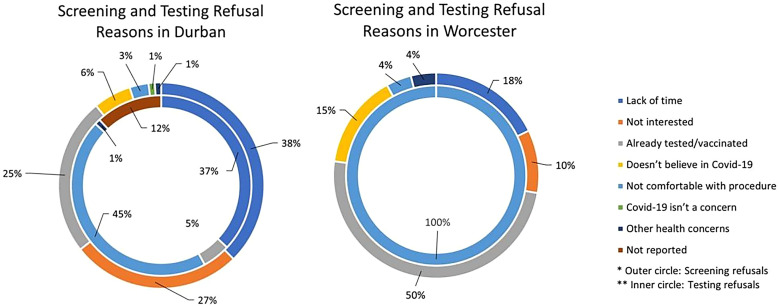
Types of refusal for coronavirus disease 2019 screening and testing by city in the randomized controlled trial.

As part of the screening pathway, individuals who refused to participate in the free COVID-19 screening and testing were asked for their reasons for refusal. Data from a total of 2,533 individuals (2,179 from Durban and 354 from Worcester) who refused indicated that participants were more likely to refuse screening because of a lack of time, disinterest, or having already been tested or vaccinated for COVID-19 in Durban (Figure 3). In Worcester, however, the primary reason for refusing screening was previous COVID-19 testing and vaccination. Among the 524 participants who agreed to be screened but refused to be tested for COVID-19, the majority were from Durban (522; 99%), and the primary reason given was discomfort with the testing procedure, with lack of time as the secondary reason for refusal. Only one person declined testing in Worcester who cited discomfort with the procedure as the primary reason for refusal.

Community perceptions regarding COVID-19 testing were analyzed through a survey of individuals who refused to participate in the RCT. A total of 704 out of the 2,533 individuals who refused agreed to take part in the survey. Notable differences were observed between participants from Durban and Worcester (Table 5). Participants from Worcester expressed a stronger consensus on the following points: COVID-19 was a public health concern (χ^2^ = 130.022; *P* <0.001), whether COVID-19 testing was an accurate and useful tool for detecting the virus (χ^2^ = 102.922; *P* <0.001), and their willingness to take their relatives to get tested if they received a positive result (χ^2^ = 2.105; df = 1; *P* >0.05).

**Table 5:** Reasons behind and willingness for COVID-19 testing of individuals from Durban and Worchester through the perception survey

Survey Questions	Response Options	Durban (*n*, %)	Worcester (*n*, %)	χ^2^ Value	df	*P*-Value
Is COVID-19 a public health concern?	Not at all	24 (35.5%)	44 (64.7%)	130.022	4	<0.001
	Somewhat	11 (52.4%)	10 (47.6%)			
	Neutral	65 (75.6%)	21 (24.4%)			
	Yes, maybe	94 (64.3%)	62 (39.7%)			
	Yes definitely	79 (21.2%)	294 (78.8%)			
Is the COVID-19 test accurate and useful in detecting the virus?	No	5 (26.3%)	14 (73.7%)	102.922	3	<0.001
	Yes	134 (32%)	285 (68%)			
	Maybe	53 (30.8%)	119 (69.2%)			
	Do not know	81 (86.2%)	13 (13.8%)			
Would you ask your family to get tested if you tested with COVID-19	No	9 (56.3%)	7 (43.8%)	2.105	1	>0.05
	Yes	264 (38.4%)	424 (61.6%)			
Reasons to test for COVID-19	Protect other people	174 (31.6%)	376 (68.4%)	–	–	–
	Requirement from work/government	139 (65.6%)	73 (34.4%)			
	It will reduce or stop COVID-19	65 (62.5%)	39 (37.5%)			
	To receive appropriate care (if sick)	39 (81.3%)	9 (18.8%)			

COVID-19 = coronavirus disease 2019; df = degree of freedom.

Age and city of residence were found to be significantly associated with people’s perceptions of the convenience of COVID-19 testing (Table 6). Younger individuals (χ^2^ = 39.749; *P* <0.01), those living in Worcester (χ^2^ = 81.261; df = 4; *P* <0.001), and males (χ^2^ = 6.71; df = 8; *P* >0.05) were more likely to perceive COVID-19 testing as convenient compared with older groups (e.g., individuals over 56 years of age had the highest proportion of disagreement), those residing in Durban, and females.

**Table 6:** Responses regarding the convenience of COVID-19 testing by age, city of residence, and sex through the perception survey

Variables		COVID-19 Testing Was a Straightforward and Convenient Process							
		Strongly Disagree (*n*, %)	Disagree (*n*, %)	Neutral (*n*, %)	Agree (*n*, %)	Strongly Agree (*n*, %)	χ^2^ Value	df	*P*-Value
Age	18–25 years	9 (6.9%)	10 (7.6%)	31 (23.7%)	51 (38.9%)	30 (22.9%)	39.749	16	<0.01
	26–35 years	11 (4.1%)	13 (4.9%)	60 (22.4%)	127 (47.4%)	57 (21.3%)			
	36–45 years	7 (4.5%)	6 (3.9%)	29 (18.8%)	52 (33.8%)	60 (39.0%)			
	46–55 years	7 (8.9%)	3 (3.8%)	19 (24.1%)	28 (35.4%)	22 (27.8%)			
	Over 56 years	10 (13.9%)	3 (4.2%)	22 (30.6%)	16 (22.2%)	21 (29.2%)			
City	Durban	9 (3.3%)	6 (2.2%)	97 (35.5%)	125 (45.8%)	36 (13.2%)	81.261	4	<0.001
	Worcester	35 (8.1%)	29 (6.7%)	64 (14.8%)	149 (34.6%)	154 (35.7%)			
Sex	Males	30 (6.9%)	17 (3.9%)	102 (23.4%)	174 (40.0%)	112 (25.7%)	6.71	8	>0.05
	Females	14 (5.3%)	18 (6.8%)	58 (21.8%)	98 (36.8%)	78 (29.3%)			
	Other	0 (0.0%)	0 (0.0%)	1 (33.3%)	2 (66.7%)	0 (0.0%)			

COVID-19 = coronavirus disease 2019; df = degree of freedom.

## DISCUSSION

This study was conducted in two cities (Durban and Worcester) in South Africa in 2022, when the overall national positivity rate of COVID-19 was low (3.6% in July 2022).^15^ However, the country has struggled with limited testing capacity since the beginning of the pandemic, which resulted in a backlog of tests, delayed results, and low confidence in public testing efforts.^4^ Despite efforts by the government to expand testing capacity and increase access to testing services, barriers to equitable testing rates across provinces and socioeconomic groups persisted because of limited testing facilities in rural areas, stigma and misinformation about COVID-19, and financial constraints.^16^ As a result of low positivity rates, our study could not provide evidence for interventions to improve isolation rates and household contact tracing in high-traffic communities. However, it did provide important information about the factors and perceptions influencing people’s willingness to test in both urban and rural settings, with implications for producing recommendations to enhance the efficacy of testing programs and targeted strategies to promote testing uptake in the local context.

Our findings indicate that age is a significant predictor of people’s willingness to test for COVID-19. Whereas younger people were more likely to perceive COVID-19 testing as convenient compared with older groups, participants over the age of 56 were almost twice as likely to consent to testing than younger populations. In South Africa, adults aged 30–39 had the highest number of COVID-19 cases in 2021, and the perceived vulnerability and higher mortality of older individuals may explain the enhanced help-seeking behavior in this population.^17^^,^^18^ Consequently, a predominantly young population (with a median age of 27.1) may account for the low national testing numbers in South Africa.^19^ This finding calls for more targeted strategies to enhance testing confidence among younger individuals. Similar to other African contexts, we also found that women were more likely to test for COVID-19 compared with their male counterparts. A study in Nigeria found that men are less likely to adopt public health behaviors or follow government actions against COVID-19 (e.g., lockdown directives). Men may exhibit lower help-seeking behaviors because of cultural and social norms, perceived susceptibility to diseases, and health literacy.^20^

In our study, the finding that individuals who had previously tested for COVID-19 (possibly during earlier high positivity waves) were less likely to re-test in the future is noteworthy. This may be due to “COVID-19 pandemic fatigue,” which suggests that efforts to maintain sustained behavior change are more challenging in contexts with prolonged restrictive measures and lockdowns. Moreover, this could reflect a perception of reduced personal risk or a belief that further testing is unnecessary after a prior positive result. In addition, it could also indicate a lack of understanding regarding the importance of repeated testing, particularly in light of new variants and waning immunity. Targeted health education campaigns may be needed to address these misconceptions and encourage regular testing, even among those with previous infection. Although there has been a global decrease in lockdown directives and mandatory face-mask rules, behavior change communication mechanisms can be used to maintain positive population, hygiene, and infection control practices to prevent future outbreaks.^21^

Since the roll-out of COVID-19 vaccines in South Africa, the country has faced a high rate of vaccine hesitancy because of misinformation and concerns about vaccine safety. In May 2022, only 45% of South Africans were fully vaccinated.^22^ Aside from the direct protection the vaccine provides to individuals and communities against infection, our data show that it might also improve willingness to test. Although the observed correlation between vaccine acceptance and testing rates may be due to inherent characteristics of the population, it is plausible that increasing population-level vaccine confidence may lead to improvements in testing rates. Thus, targeted efforts to improve population-level vaccine confidence could potentially have a spillover effect on promoting testing behaviors within the population.

The psychosocial responses of populations during an infectious disease outbreak are crucial in influencing the transmission of the disease, as well as the incidence of emotional distress and the impact on social capital both during and after the outbreak. However, despite the importance of psychological factors, resources allocated toward exploring their impact on systems-level detection and management remain neglected and inadequate. We found a disproportionately large burden of depression and anxiety symptoms in the study population, with 52% of the participants reporting mild symptoms and 16% reporting moderate to severe symptoms, which is comparable to estimates from other local research studies.^23^ Although there is some evidence in high-resource settings indicating that individuals with psychosocial distress may be less likely to test,^24^ we did not find a significant association between willingness to test and the severity of depression or anxiety symptoms in our population. However, this may not be universal across the wider population or context. Given the high burden of mental health distress in the population, there is a need to invest in larger-scale implementation research to better understand the interplay between psychosocial factors and COVID-19 testing behaviors in different populations within South Africa.

Although local evidence suggests that rural townships were the hardest hit during the government’s hardline response to COVID-19 lockdowns,^25^ our study showed negligible refusals for COVID-19 testing and higher perceived confidence in the protective factors of COVID-19 self-testing and family referrals in rural populations. Approximately 68% of participants in rural Worcester strongly agreed that COVID-19 was a serious public health concern, compared with 29% in Durban. This significant difference in refusal rates is an important consideration, suggesting that there may be contextual factors, such as local norms, access to healthcare, or trust in public health authorities, that influence testing behaviors in different communities. Further qualitative research would be valuable to explore the reasons behind these geographic differences.

### Limitations.

This study has several limitations. First, the data were collected during the later stages of the COVID-19 pandemic, when many public health restrictions had been relaxed in South Africa. This may have influenced people’s perceptions and behaviors regarding testing. In March 2022, South Africa lifted most of its COVID-19 restrictions, including the requirement for masks in public places and the limitation on gatherings. However, some measures, such as testing and contact tracing, continued. In April 2022, the South African government announced the end of the National State of Disaster, marking a significant shift toward normalcy. In May 2022, despite the relaxation of restrictions, the Department of Health emphasized the importance of testing, contact tracing, and vaccination. From June to December 2022, the country experienced a period of relatively low case numbers and hospitalizations, which may have influenced public perception and behavior regarding COVID-19 testing and vaccination.

The relaxed restrictions and the general decrease in COVID-19 cases during the study period may have influenced people’s perceptions and behaviors regarding testing. For instance, individuals might have been less motivated to undergo testing because of a perceived lower risk of infection. Additionally, this study’s findings on testing uptake and mental health symptoms should be considered in the context of this period. The reduced sense of urgency and fear of infection could have affected individuals’ willingness to participate in testing and the reported mental health outcomes. These limitations should be taken into account when interpreting the study’s findings.

## CONCLUSION

This study explored the factors influencing COVID-19 testing uptake in urban and rural South African communities, offering valuable insights into the complex interplay of sociodemographic, psychological, and contextual factors that shape testing behaviors. Our findings indicate a need for investment in larger-scale implementation research to better understand the impact of targeted information campaigns on COVID-19 testing behaviors across different populations in South Africa.
